# The Jewish Hospital, Manchester

**Published:** 1911-10-28

**Authors:** 


### THE JEWISH HOSPITAL, MANCHESTER.

### Its Interesting and Peculiar Features.

At the recent Conference held in Manchester the members of the British Hospitals Association received an invitation from one of the Vice-Presidents (Mr. Julius I. Loewy) of the Victoria Memorial Hospital to visit that institution. We accepted that invitation and paid an interesting although somewhat hurried visit. The hospital is situated in the Jewish quarter of the city—but it is unnecessary to mention this, as the facial characteristics of the inhabitants will be ample evidence of the fact. Mr. A. Cardy, the Secretary, placed himself very courteously at our disposal, and the Matron (Miss M. Cash) also assisted in doing the honours of the occasion. Before dealing with the internal arrangements of the hospital a few words are necessary as to its foundation.

It is claimed that this is the only Jewish General Hospital in the United Kingdom, and the scheme for its foundation seems to have first been mooted in 1900, when, to quote from the first report of the Hospital, "a number of earnest workers, impelled by the great need for such an institution where their sick poor shall be able to obtain during periods of illness Kosher food, and have the consolation of seeing Jewish faces around them, privileges appealing very strongly to the suffering poor, banded themselves together, and, after several meetings held at the Kovner Synagogue, decided to convene a conference, which was held at the Jewish Working Men's Club in July 1900, to which many leading members of the community were invited, and a discussion ensued as to the best method of bringing the project to a successful issue." Dr. Charles Dreyfus, J.P., a City Councillor, was appointed president, and under his guidance negotiations proceeded. In 1901 a public meeting, held in the Memorial Hall, Sir W. H. Bailey presiding, resolved to pledge itself to support the founding of the Manchester Victoria Memorial Jewish Hospital.

This resolution was put into effect and the hospital reared itself upon the site selected in 1904—the opening ceremony taking place on November 17 in that year. The hospital and its equipment can therefore claim to be the outcome of the enterprise of the Jewish community of the district.

The hospital is maintained by the usual subscriptions and donations, but there is an unusual feature, *i.e.* that two-fifths of the subscriptions are by weekly payments. The work of collecting these sums devolves upon a band of some sixty honorary workers, who make over two thousand calls per week, and it seems characteristic of the management that they do not despise the smaller coins of the realm, as their first appeal for subscriptions was one for a million pennies. Another peculiar item in the income is that relating to weddings and engagements, but although the list of contributions under these heads is somewhat lengthy, the amount received is comparatively small, the logical conclusion being that the financial status of those Jews who have recently married is not so high as one would expect.

Turning to the administration, another unusual procedure is that the Board of Management holds its meetings on a Sunday. It is the only hospital we know of where Sunday is selected for board meetings, but then of course Saturday is observed as Sabbath in this hospital.

Although the hospital is primarily for the Jews, patients of other religious beliefs are not excluded, and on the day of inspection there were as many of the Christian as of the Jewish religion occupying the beds. Although the regulations are framed solely for the Jewish community, they do not appear to be irksome to others, many of whom seem to prefer this little hospital to its more pretentious neighbours, possibly on the score of its homeliness.

The wards, which contain some forty beds and cots, are light and airy, and cleanliness is evident. A visit to the kitchens revealed that the cooking was entirely Jewish in character, except in the pantry, where one found delicacies for which it was a little difficult to account, until it appeared that the Matron, who is also instructress to the cook, is a gold medallist in this art. There are large cellars or basement rooms used for storage purposes, and kept in a state of spotless cleanliness; but why so much space should be allocated for cellars when it could be perhaps utilised better in the more essential parts of the building appears to need some explanation.

The management is fortunate in having as its Secretary a gentleman who has been trained in the office of Mr. Walter G. Carnt, the Secretary of the Royal Infirmary, Manchester. Mr. Cardy has been there only a few months, but from what little we saw of his work we feel sure he will justify expectations.

The management appears careful in its expenditure, both of capital and revenue, but a comparison of the usual heads of expenditure is impossible owing to the absence from the report of the tables of statistics as to patients, etc., and to the accounts having been compiled under a classification not in use amongst hospitals generally. The Board appears to be particularly anxious as to the custody of their moneys at the bank, as their regulations require that all cheques be signed by three members of the Board, or by the Treasurer and two members; but this carefulness is possibly only a national trait.

The Trustees and Board of Management possess a hospital which compares favourably with others of its size throughout the country, and they are to be congratulated on their pluck in tackling the scheme with the limited funds at their disposal, and their action in admitting Gentile as well as Jew to their wards is instructive, seeing that the hospital is separatist in origin.

